# Medicaid Payments and Outcomes for Pediatric Dental Surgical Procedures by Site of Care

**DOI:** 10.1001/jamanetworkopen.2025.37081

**Published:** 2025-10-10

**Authors:** Ashley M. Kranz, Xiaoxi Zhao, Elizabeth Munnich, Jessica Y. Lee, Christopher Whaley

**Affiliations:** 1 RAND Corporation, Arlington, Virginia; 2 RAND Corporation, Santa Monica, California; 3 University of Louisville, Louisville, Kentucky; 4 University of North Carolina Adams School of Dentistry, Chapel Hill; 5 Brown University School of Public Health, Providence, Rhode Island

## Abstract

**Importance:**

Despite need, children face access barriers to dental surgical procedures. Surgical procedures for older adults delivered in ambulatory surgical centers (ASCs) are less costly than and of comparable quality to care delivered in hospital outpatient departments (HOPDs), but it remains unknown whether this association extends to pediatric dental surgical procedures.

**Objective:**

To determine whether payment, time from diagnosis to care, and adverse events for pediatric surgical procedures for dental caries vary between ASCs and HOPDs paid by Medicaid.

**Design, Setting, and Participants:**

This cross-sectional study of Medicaid data (July 1, 2016, through January 31, 2020) used an instrumental variables regression approach and exploited a source of exogeneous variation influencing ASC supply. Analysis was conducted October 2024 to April 2025, using records from 29 states with high-quality Medicaid data. Participants were children aged 18 years or younger enrolled in Medicaid who underwent surgical procedures for dental caries in an ASC or HOPD.

**Exposure:**

Receipt of dental surgical care in an ASC or HOPD.

**Main Outcomes and Measures:**

The primary outcomes were Medicaid payment, days from diagnosis to surgery, and any emergency department visits and hospitalizations within 7 days after surgery.

**Results:**

Across 391 628 pediatric dental surgical procedures, patients had a mean (SD) age of 5.00 (3.05) years, and 211 819 (54.09%) were male. Medicaid payment was $604 (95% CI, $149-$1058; *P* < .009) less for a surgery occurring in an ASC vs an HOPD, corresponding to a 27% reduction in spending, and time from diagnosis to surgery was 8.9 days (95% CI, 5.5-12.3 days; *P* < .001) less for a surgery occurring in an ASC than HOPD. A surgery occurring in an ASC (vs HOPD) was statistically significantly less likely to be followed by an emergency department visit or hospitalization within 7 days after surgery (0.27 percentage points; 95% CI, 0.18-0.36 percentage points) and a hospitalization within 30 days after surgery (0.28 percentage points, 95% CI, 0.11-0.45 percentage points).

**Conclusions and Relevance:**

In this cross-sectional study of pediatric surgical procedures for dental caries paid by Medicaid, procedures performed in ASCs were less costly and had lower rates of adverse events compared with those performed in HOPDs. The time from diagnosis to surgery was shorter for surgical procedures in ASCs compared with HOPDs, suggesting that ASCs may improve access to care for children from low-income families needing for dental treatment. Increased use of ASCs for pediatric dental surgical procedures may reduce costs for Medicaid and improve patient experiences.

## Key Points

**Question:**

Do payments, time from diagnosis to surgery, and adverse events vary for pediatric dental surgical procedures delivered in ambulatory surgical centers (ASCs) and hospital outpatient departments (HOPDs) paid by Medicaid?

**Finding:**

In this cross-sectional study of 391 628 pediatric surgical procedures for dental caries, surgical procedures in ASCs occurred significantly more quickly after diagnosis than those in HOPDs. Surgical procedures in ASCs were statistically significantly less costly and had significantly lower adverse event rates than HOPDs.

**Meaning:**

These findings suggest that Medicaid programs can save money and increase timely delivery of care by expanding use of ASCs for pediatric dental surgical procedures.

## Teaser

This cross-sectional study compares Medicaid payments, time from diagnosis to care, and adverse events for pediatric surgical procedures for dental caries and performed in ambulatory surgical centers vs hosptal outpatient departments.

## Introduction

Oral health disparities for children are substantial, with high rates of dental caries, commonly called tooth decay, among children from low-income households.^1,2^ Untreated caries is associated with pain and decreased quality of life,^3,4,5^ increased risk of future caries,^6,7^ missed days of school,^8^ poor school performance,^8^ and, in extreme cases, death.^9^ The greater the burden of disease, the more likely it is for treatment to be provided in a surgical setting using general anesthesia (GA). GA may be indicated for patients with extensive treatment needs, complex treatment plans, uncooperative behavior, severe anxiety or fear, physical or mental challenges, and for patients who are very young.^10^ Although these procedures are rare−accounting for approximately 27 procedures per 10 000 Medicaid enrollees in 2019^11^−their frequency varies by state and they may contribute to 25% of a state’s annual Medicaid dental spending or up to 2% of a state’s annual Medicaid spending on children.^12,13^ Despite need, children face access barriers to dental surgical procedures. A national study of pediatric dentistry residency program directors found that a child in pain waits an average of 28 days for dental treatment under GA, and that some children wait up to 5 months.^14^ A survey of children’s hospitals indicated the access to operating rooms for dental procedures has declined since 2017, leading to longer wait times.^15^

Pediatric dental surgical procedures requiring the use of GA are typically outpatient procedures conducted in ambulatory surgical centers (ASCs) and hospital outpatient departments (HOPDs). Free-standing ASCs operate outside of a traditional hospital. Research, primarily on older adults, suggests that surgical procedures conducted in ASCs are less costly and have better or comparable quality and patient satisfaction as procedures conducted in HOPDs,^16,17,18,19,20,21^ although some types of surgical procedures paid by commercial insurers may be more costly in ASCs.^22,23^ Surgical procedures take less time in ASCs than HOPDs, which may contribute cost savings.^18,24,25^ These studies, however, largely focused on older adults and did not examine dental care or pediatric populations. The few studies that have examined pediatric dental surgical procedures have found increasing use of ASCs and lower costs in ASCs than HOPDs. Sheen and colleagues^11^ reported increasing rates of pediatric dental surgical procedures paid by Medicaid in ASCs compared with relatively flat rates in HOPDs from 2019 to 2021. Lee and colleagues^12^ examined pediatric dental surgical procedures paid by Medicaid in 7 states from 2011 to 2012 and found that average payments were approximately $560 lower in ASCs than HOPDs. Grisel and colleagues^17^ examined 147 pediatric dental surgical procedures at a medical center in 2008 and found that their ASC (vs HOPD) was less costly and more efficient. These studies, however, were conducted over 10 years ago and did not adjust for underlying factors that may be associated with receipt of care in an ASC. Comparing cost and outcomes for procedures in ASCs and HOPDs is challenging due to the potential for selection bias. If ASCs treat healthier patients on average, then any observed differences in outcomes may be associated with differences in patient composition across settings rather than differences in quality of care.

This study expands on prior research by using Medicaid data from 29 states during the period from 2016 to 2020, exploiting a source of exogeneous variation influencing ASC supply to address the potential for selection bias, and exploring several outcomes of interest to payers and patients. The objective of this study was to compare payments, time from diagnosis to care, and adverse events for pediatric surgical procedures for dental caries in ASCs and HOPDs paid by Medicaid. To mitigate concerns about selection bias, we used an instrumental variables (IV) estimation approach using state-level certificate-of-need (CON) laws.

## Methods

### Data

This cross-sectional study used the 2016 to 2020 Transformed Medicaid Statistical Information System Analytic Files, which provide information about individuals enrolled in Medicaid in each state, as well as dental and medical care they receive. Information about state CON laws was obtained from the National Conference on State Legislatures.^26^ CON laws are state-level regulatory mechanisms designed to oversee major capital investments in health care facilities, and to control costs by preventing unnecessary duplication of services and ensuring that new expenditures align with community needs. In states with CON laws, a designated health planning agency or regulatory body must approve the establishment of new health care facilities or the expansion of existing services within a specified area. Both additional barriers and potential capture of CON boards by existing facilities and services can deter entry or expansion of new facilities and services. The Box lists the states have CON laws and whether these laws specifically include ASCs. This study follows Strengthening the Reporting of Observational Studies in Epidemiology (STROBE) reporting guideline for cross-sectional studies and was deemed exempt by RAND’s human participants’ protection committee (category 4—secondary research for which consent is not required).^27^

Box. **List of States with Certificate-of-Need (CON) Laws and States Included in this Analysis**

**15 States Without a CON Law**

Arizona
California
Colorado^a^
Idaho
Kansas^a^
Minnesota
New Hampshire^a^
New Mexico
North Dakota
Pennsylvania
South Dakota^a^
Texas^a^
Utah^a^
Wisconsin
Wyoming^a^

**26 States With an ASC-Specific CON Law**

Alabama^a^
Alaska^a^
Connecticut^a^
District of Columbia^a^
Delaware^a^
Georgia^a^
Hawaii
Illinois
Iowa^a^
Kentucky^a^
Maine
Maryland^a^
Massachusetts
Michigan
Mississippi^a^
Nevada^a^
New Jersey
New York
North Carolina^a^
Rhode Island
South Carolina^a^
Tennessee^a^
Vermont
Virginia
Washington^a^
West Virginia

**10 States With a CON Law Not Specific to ASCs**

Arkansas
Florida
Indiana^a^
Louisiana^a^
Missouri^a^
Montana
Nebraska^a^
Ohio^a^
Oklahoma^a^
Oregon^a^

Abbreviation: ASC, ambulatory surgery center.

^a^
Identifies the 29 states included in our main analysis.

### Sample

We identified outpatient surgical procedures in ASCs and HOPDs with a diagnosis of dental caries (*International Statistical Classification of Diseases and Related Health Problems, Tenth Revision* diagnostic code K02) (eTable 1 in Supplement 1). We examined children aged up to 18 years with full Medicaid benefits. To allow for examination of dental visits occurring before and after the procedure, we required 8 months of continuous enrollment (6 months prior and 2 months following each procedure) and limited our sample to procedures occurring from July 1, 2016, to January 31, 2020, to exclude the emergence of COVID-19. Because concerns about Transformed Medicaid Statistical Information System Analytic File data quality are well documented,^28,29,30,31^ we excluded state-years deemed unusable or of high concern by the Data Quality Atlas,^29^ if place of service was unknown for more than 20% of dental caries procedures in a state-year, and if more than 20% of procedures were missing a dental diagnosis within 6 months before the surgical procedure.

### Outcomes

We examined 4 outcomes: Medicaid payment, days from diagnosis to procedure, emergency department (ED) visit within 7 days of the procedure, and hospitalization within 30 days of the procedure. Total Medicaid payment was calculated by summing all fee-for-service (FFS) paid amounts on the date of the procedure. This was calculated only for FFS procedures due to concerns about inaccurate and missing information on procedures paid via managed care (31.4% of procedures examined). We adjusted payments to 2016 dollars using the Consumer Price Index for Medical Care and excluded extreme values (top and bottom 1% of the payment distribution). We measured time from diagnosis to procedure, up to 6 months prior, by counting back the number of days from the procedure to the most recent encounter or claim with a diagnosis of dental caries in the outpatient claims file. Because dentists typically use procedure codes rather than diagnosis codes when billing dental insurers, we searched for the most recent occurrence of a procedure codes indicating evaluation and diagnosis or diagnosis of dental caries or diseases of pulp and periapical tissues (codes listed in eMethods in Supplement 1). We examined claims for ED visits for any reason within 7 days, excluding the day of the procedure, and any hospitalization within 30 days, including the day of the procedure (codes listed in eMethods in Supplement 1).

### Statistical Analysis

Data analysis was conducted from October 2024 to April 2025. We produced descriptive statistics (means, SDs, and percentages) to summarize the outcomes and characteristics of patients by place of service. Estimating the association between the site of care, Medicaid payment, and patient outcomes is challenging using administrative data because patients and their families, with input from their practitioners, self-select into sites of care. Because this selection is unobserved, differences in patient outcomes and payments may be biased, even after controlling for observed factors. For example, a patient’s risk status could be associated with both the choice of site of care and with costs or outcomes. A patient with a high (but unobservable in administrative data) risk of complications may prefer to obtain care in an HOPD, and therefore we may observe higher costs related to this risk status.

To mitigate this type of selection bias, we exploited a source of exogeneous variation influencing ASC supply. Specifically, we implemented an IV estimation approach using a binary indicator of whether a state has a CON law. CON laws regulate the size of existing facilities and the building of new facilities. Our use of CON laws as an IV requires 2 assumptions. First, there should be a correlation between CON laws and an individual’s choice of setting of care (eg, the first-stage assumption that the instrument estimates the endogenous treatment). To test this first requirement, we calculated an *F* statistic based on the results of the first-stage regression. Our *F* statistic was 1108, which is much larger than the commonly used threshold of 10, suggesting that our IV was highly correlated with the endogenous variable, the site of care.

The second required assumption is that the IV will not influence outcomes outside of its influence on patient’s receipt of care in an ASC vs HOPD (exclusion restriction). Although this assumption is fundamentally untestable, conceptually CON laws—state-level laws regulating supply side changes—are unlikely to be correlated with an individual patient’s outcomes when the place of service is chosen. Importantly, most state decisions on CON policies were determined several decades ago. Thus, current patient access to ASC and HOPD practitioners is unrelated to historical decisions to implement a CON law.

We estimated the IV model using a control function-based 2-stage residual inclusion because the dependent variable in the first-stage model was a dichotomous indicator of the surgical procedure occurring in an ASC (vs HOPD).^32^ We implemented a logit-regression at the first stage and a generalized linear model at the second stage with log-form link function. The models controlled for age, sex, race and ethnicity, number of dental codes associated with a claim, a dichotomous measure of pediatric surgical risk, and year fixed effects. The racial and ethnic categories included in the model were obtained drectly from the TAF data and are listed in Table 1. Race and ethnicity was controlled for because prior research has found differential access to ASCs and HOPDs by race and ethnicity for pediatric dental surgical procedures and other surgical procedures.^11,33^ The number of dental codes can be considered a measure of disease severity because having more codes indicates that more treatment was received. We used a risk score that identifies risk of mortality, which uses information on age, sex, and diagnosis codes.^34^ The diagnosis codes reflect 69 condition categories from the Agency for Healthcare Research and Quality’s Clinical Classifications Software Refined.^35^ Although the score can range from 0 to 6 or more, we created a dichotomous indicator of low risk (score = 0) or some risk (score >0) because greater than 95% of children undergoing a procedure had a score of 0.

**Table 1.** Characteristics of Pediatric Dental Surgical Procedures Paid by Medicaid Furnished in ASCs and HOPDs, 2016-2020

Variable	Patients, No. (%)		
	All (N = 391 628)	ASC (n = 117 625)	HOPD (n = 274 003)
Outcomes			
Time from diagnosis to surgery, mean (SD), d	53.25 (41.62)	46.91 (38.91)	55.98 (42.44)
Emergency department visit within 7 d of surgery, mean (SD), %	1.70 (12.93)	1.40 (11.79)	1.80 (13.39)
Hospitalization within 30 d of surgery, mean (SD), %	0.33 (5.76)	0.15 (3.89)	0.41 (6.40)
Medicaid payment, mean (SD), $^a^	2209 (1298)	1835 (829)	2365 (1420)
Characteristics of sample			
Age, mean (SD), y	5.00 (3.05)	4.86 (2.86)	5.06 (3.12)
Sex			
Female	179 809 (45.91)	54 248 (46.12)	125 561 (45.82)
Male	211 819 (54.09)	63 377 (53.88)	148 442 (54.18)
Race and ethnicity^b^			
American Indian and Alaska Native, non-Hispanic	10 843 (2.77)	4622 (3.93)	6221 (2.27)
Asian, non-Hispanic	6672 (1.7)	1906 (1.62)	4766 (1.74)
Black, non-Hispanic	65 399 (16.7)	14 371 (12.22)	51 028 (18.62)
Hawaiian or Pacific Islander	2001 (0.51)	629 (0.53)	1372 (0.50)
Hispanic, all races	90 964 (23.23)	37 600 (31.97)	53 364 (19.48)
White, non-Hispanic	119 372 (30.48)	28 175 (23.95)	91 197 (33.28)
Missing or unknown	92 918 (23.73)	29 545 (25.12)	63 373 (23.13)
Multiracial, non-Hispanic	3459 (0.88)	777 (0.66)	2682 (0.98)
Risk score			
No risk	379 894 (97.00)	114 092 (97.00)	265 802 (97.01)
Some risk	11 734 (3.00)	3533 (3.00)	8201 (2.99)
Count of dental procedure codes, mean (SD)	15.32 (8.47)	15.82 (7.79)	15.10 (8.74)

Abbreviations: ASC, ambulatory surgery center; HOPD, hospital outpatient department.

^a^ Payment is presented in 2016 US dollars and limited to 122 832 fee-for-service surgical procedures, of which 29% were in ASCs.

^b^ We used the race and ethnicity categories and variable labels provided in the Medicaid Transformed Medicaid Statistical Information System analytic file data.

To assess the sensitivity of our results to different samples, we also estimated models using a sample restricted to balanced panels of 11 states during 2016 to 2020 and 15 states during 2017 to 2020. We used Stata/MP statistical software version 17.0 (StataCorp) for analyses. A 2-sided significance threshold was set at *P* < .05 with Wald tests used to assess statistical significance. We present coefficient estimates for linear regressions and marginal effects for logit regressions, with SEs clustered at the state level.

## Results

After exclusions for quality concerns and missing information, we examined 391 628 surgical procedures for dental caries in ASCs and HOPDs in 29 states (eTables 2 and 3 in Supplement 1). Patients had a mean (SD) age of 5.00 (3.05) years, and 211 819 (54.09%) were male. Unadjusted results are presented in Table 1. The mean (SD) number of days from diagnosis to surgical procedure was 46.91 (38.91) days for procedures in ASCs and 55.98 (42.44) days for procedures in HOPDs. Rates of postsurgery ED visits or hospitalizations were similar across settings. Mean (SD) FFS Medicaid payments were lower in ASCs than HOPDs ($1835 [$829] vs $2365 [$1420]). The characteristics of patients who underwent procedures in ASCs and HOPDs were generally similar.

Adjusted associations between ASC use and the outcome variables are presented in Table 2. Compared with HOPD use, ASC use was associated with receiving care sooner and small declines in the probability of a postsurgery ED visit or hospitalization. ASC use was associated with statistically significantly lower mean Medicaid FFS payments (mean [SE], $557 [$187]; *P* = .006).

**Table 2.** Adjusted Regression Results Showing Association Between Receipt of Care in an ASC on Time to Care, Adverse Outcomes, and Payments for Pediatric Dental Surgical Procedures Paid by Medicaid, 2016-2020

Regression variables	Days from diagnosis to surgery	ED visit within 7 d of surgery, %	Hospitalization within 30 d of surgery, %	Medicaid payment, $^a^
Ordinary least squares				
Mean (SE)	−8.53 (1.59)	−0.004 (0.0005)^b^	−0.003 (0.0009)^b^	−557 (187)
*P* value	<.001	<.001	.001	.006
Instrumental variables estimates				
Mean (SE)	−8.94 (1.74)	−0.0027 (0.0005)	−0.0028 (0.0009)	−604 (232)
*P* value	<.001	<.001	.002	.009
Relative changes, % (95% CI)	−16.80 (−23.17 to 10.39)	−16.00 (−21.21 to 10.83)	−84.10 (−136.48 to 31.79)	−27.30 (−47.91 to 6.77)
No. of observations	391 628	391 628	391 628	122 832

Abbreviations: ASC, ambulatory surgery center; ED, emergency department.

^a^ Sample is limited to surgical procedures paid fee-for-service.

^b^ Results are marginal effects from logistic regressions.

Because of concerns that associations between ASC use and outcomes may be due to patient selection, we implemented an IV strategy using an indicator that a state had any CON laws. As shown in Table 2 and eTable 4 in Supplement 1, results of the IV regressions found that ASC use was associated with a shorter mean (SD) time from diagnosis to surgery by 8.9 days (95% CI, 5.5-12.3 days; *P* < .001), which corresponds to a relative decrease of 16.8%. ASC use was also associated with a lower probability of having an ED visit within 7 days of surgery by 0.27 percentage points (95% CI, 0.18-0.36 percentage points; 16.0% relative decrease) and a lower probability of being hospitalized within 30 days of surgery (0.28 percentage points; 95% CI, 0.11-0.45 percentage points; 84.1% relative decrease). ASC use was associated with decreased Medicaid FFS payment for a procedure by a mean of $604 (95% CI, $149- $1058). This represents a 27.3% relative decrease in Medicaid FFS payment. Using the IV regression estimates, Medicaid would be projected to save $53.6 million if all pediatric surgical procedures for dental caries for the Medicaid FFS population were furnished in ASCs. Assuming similar costs for Medicaid managed care enrollees, the projected total savings for Medicaid would increase to $171 million. Sensitivity tests using balanced panels of states show similar results to our main findings (eTable 5 in Supplement 1).

## Discussion

This cross-sectional study used Medicaid data from 29 states to compare outcomes for pediatric surgical procedures for dental caries furnished in ASCs and HOPDs. We found that ASC use was associated with a shorter time from diagnosis to surgery, lower rates of postsurgery ED visits and hospitalizations, and lower Medicaid payments.

Children insured by Medicaid and needing surgery for dental caries typically wait a long time for care. In our study, the mean time from diagnosis to surgery was nearly 2 months, and a national survey reported that families may wait up to 5 months,^14^ with wait times increasing for HOPDs.^15^ Receiving care more quickly is important because of the pain associated with untreated dental caries, which contributes to decreased quality of life,^3,4,5^ missed days of school,^8^ and poor school performance.^8^ We found that ASC use decreased the waiting time from diagnosis to surgery by more than 1 week, without increasing adverse events. This finding highlights that expanding where care is delivered can help to increase access to care for Medicaid enrollees.

Our finding that ASC use is associated with lower costs aligns with prior research examining pediatric dental surgical procedures^12,17^ and adult outpatient surgical procedures.^16,17,18,19,20,21^ Greater use of ASCs for pediatric dental surgical procedures may result in cost-savings for state Medicaid programs, estimated at an upper bound of approximately $171 million. Other insurers have recognized the potential cost-savings of ASCs and enacted policies to encourage ASC use. For example, the California Public Employees’ Retirement System implemented a reference pricing model, in which eligible patients who receive care from a HOPD were responsible for paying price differentials, leading to a 21% savings, with no meaningful change in complications.^36^ If programs like this expand, future studies could explore impacts to HOPD revenue if more procedures move to ASCs.

Despite the potential for cost-savings, there are a variety of factors that influence the setting of care for these procedures. Importantly, only approximately 8% of ASCs offer dental care.^37^ If a child has access to both an ASC and HOPD, determining where the surgery occurs is a decision largely made by the clinical team, which is informed by their preferences, relationships, and the underlying health of the patient. Additionally, patient and caregiver preferences influence when and where the procedure occurs. Some may feel more comfortable receiving care at an HOPD, where there is easy access to emergency services and translation services may be more accessible.

While cost-savings is important, Medicaid programs are required to provide access to medically necessary pediatric dental care and that may mean paying more for care.^38^ Effective January 1, 2024, the Centers for Medicare & Medicaid Services (CMS) increased Medicare payments to both HOPDs and ASCs for dental surgical procedures with the goal of incentivizing increased availability of operating rooms for these procedures. This was motivated by reports about long wait times for pediatric dental surgical procedures at children’s hospitals^15^ and concerns from pediatric dentists that HOPDs deny access to pediatric dental procedures due to financial pressures, because dental care is typically less profitable than medical care due to lower facility fees.^39^ Although Medicaid programs are not required to follow this CMS rule, Rhode Island’s Medicaid program has adopted a similar increase to improve access to care for dental surgical procedures.^40^ CMS authorized Medicare payment to hospitals and ASCs for dental care at $3070.81 and $1318.93, respectively, indicating that ASCs are likely to remain at least half as costly as HOPDs.^39^

### Limitations

This study has limitations that should be mentioned. First, known concerns about Transformed Medicaid Statistical Information System Analytic Files data quality led us to exclude many state-years of data,^28,29,30,31^ meaning our findings may not be generalizable to all states. Second, because ours is not a randomized clinical trial, we can only speculate about the causal relationship between ASC use and the outcomes examined, although our IV approach brings rigor to our analysis. Third, our IV does not vary within states over time, so we were unable to include state fixed effects in these models. Future analyses should consider approaches to causal inference that allow for the inclusion of state fixed effects. Fourth, although we constructed our risk score using diagnoses occurring up to 6 months before the procedure, claims data lack clinical detail and may not capture all medical comorbidities. Fifth, although we examined a variety of outcomes, additional research is needed to examine patient-centered outcomes, including distance to care and satisfaction. Sixth, due to concerns about inaccurate and missing information on services paid via managed care, we examined only FFS Medicaid payments. In our sample, only 31.4% of surgical procedures were paid FFS, and more than 80% of children are enrolled in managed care in 36 states.^41^ Lacking information on payments by Medicaid managed care plans, we cannot be sure that ASC use will lead to cost-savings for these plans. However, other studies have also found that ASC use is less costly for other procedures paid by Medicare and commercial insurers.^16,17,18,19,20,21^

## Conclusions

In this cross-sectional study of pediatric dental surgical procedures paid by Medicaid, ASC use was associated with a shorter time from diagnosis to surgery, lower rates of postsurgery ED visits and hospitalizations, and lower Medicaid payments than HOPDs. Expanded use of ASCs for these outpatient surgical procedures could save Medicaid agencies money and increase timely receipt of care.

## Notes

Supplement 1. **eMethods.** Additional details about the dataset, sample, and variables **eTable 1.** Codes to identify surgical procedures in ASCs and HOPDs **eTable 2.** Exclusion criteria table **eTable 3.** States and years included in analysis **eTable 4.** Estimated coefficients from IV model **eTable 5.** Sensitivity test results: Instrumental Variable estimates of effects of receipt of care in an ASC on time to care, adverse outcomes, and payments for pediatric dental surgeries paid by Medicaid

Supplement 2.
Data Sharing Statement
