# Supplementary material for: Medicaid Payments and Outcomes for Pediatric Dental Surgical Procedures by Site of Care
Source: JAMA Netw Open. 2025 Oct 10;8(10):e2537081. doi: 10.1001/jamanetworkopen.2025.37081 (PMC12514629; doi:10.1001/jamanetworkopen.2025.37081)
Supplement: Supplement 1. — eMethods. Additional details about the dataset, sample, and variables eTable 1. Codes to identify surgical procedures in ASCs and HOPDs eTable 2. Exclusion criteria table eTable 3. States and years included in analysis eTable 4. Estimated coefficients from IV model eTable 5. Sensitivity test results: Instrumental Variable estimates of effects of receipt of care in an ASC on time to care, adverse outcomes, and payments for pediatric dental surgeries paid by Medicaid [file jamanetwopen-e2537081-s001.pdf]

Supplemental Online Content

Kranz AM, Zhao X, Munnich E, Lee JY, Whaley C. Medicaid payments and outcomes for pediatric dental operations by site of care. *JAMA Netw Open.* 2025;8(10):e2537081. doi:10.1001/jamanetworkopen.2025.37081

eMethods 1. Additional details about the dataset and samples

eMethods 2. Additional details about variables

eTable 1. Codes to identify surgical procedures in ASCs and HOPDs

eTable 2. Exclusion criteria table

eTable 3. States and years included in analysis

eTable 4. Estimated coefficients from IV model

eTable 5. Sensitivity test results: Instrumental Variable estimates of effects of receipt of care in an ASC on time to care, adverse outcomes, and payments for pediatric dental surgeries paid by Medicaid

This supplemental material has been provided by the authors to give readers additional information about their work.

eMethods 1. Additional details about the dataset and sample

This study used the 2016-2020 Transformed Medicaid Statistical Information System (T-MSIS) Analytic Files (TAF), which provide information about individuals enrolled in Medicaid in each state as well as dental and medical care they receive. We used the Demographic and Eligibility File to identify enrollee characteristics and enrollees with full Medicaid benefits, the Other Services File to identify dental caries diagnosis, outpatient surgical procedures, emergency department (ED) visits, and costs of surgical procedures, and the Inpatient File to identify hospitalizations.

We identified outpatient surgical procedures in ASCs and HOPDs using the place of service variable with a diagnosis of dental caries (International Classification of Diseases tenth revision code (ICD-10) K02). If the place of service was missing, we used other codes listed in Appendix Table A1.

© 2025 Kranz AM et al. *JAMA Netw Open*.

eMethods2. Additional details about variables

We measured time from diagnosis to procedure, up to six months prior, by counting back the number of days from the procedure to the most recent encounter or claim with a diagnosis of dental caries in the outpatient claims file. Because dentists typically use Current Dental Terminology (CDT) codes rather than diagnosis codes when billing dental insurers, we searched for the most recent occurrence of a procedure codes indicating evaluation and diagnosis (CDT codes D0120, D0140, D0145, D0150, D0160, D0190, D9310) as well as diagnosis of dental caries or diseases of pulp and periapical tissues (ICD-10 codes K02 and K04).

We examined claims for ED visits for any reason within 7 days, excluding the day of the procedure, using revenue code (0450, 0451, or 0452), place of service (23), or procedure code (Current Procedural Terminology codes 99281, 99282, 99283, 99284, 99285).

© 2025 Kranz AM et al. *JAMA Netw Open*.

eTable 1. Codes to identify surgical procedures in ASCs and HOPDs

	**Ambulatory surgery center (ASC)**	**Hospital outpatient department (HOPD)**
Place of Service	24	22 and 19
When place of service was missing, used:		
Bill Type Code	083x	013x
Billing Provider Type Code	13	
Billing Provider Taxonomy Code	261QA1903X	282N00000X, 282NC2000X, 281PC2000X, 282NC0060X, 282NR1301X
Type of Service		002

© 2025 Kranz AM et al. *JAMA Netw Open*.

eTable 2. Exclusion criteria table

	N observations	N observations dropped
Initial sample	3,804,662	
Drop if episode does not occur in ASC or HOPD	993,800	2,810,862
Drop if episode occurs in state-year with poor quality data	527,964	465,836
Drop if index date occurs after February 1, 2020	446,410	81,554
Drop if missing time from initial diagnosis to surgical procedure	393,770	52,640
Drop if missing information on sex	393,761	9
Drop if missing count of dental codes	391,628	2,133

Note. Abbreviations: ASC, ambulatory surgery center. HOPD, hospital outpatient department.

© 2025 Kranz AM et al. *JAMA Netw Open*.

eTable 3. States and years included in analysis

	2016	2017	2018	2019	2020
**AK**			X	X	
**AL**		X	X		X
**CO**	X			X	X
**CT**	X	X	X	X	X
**DC**	X		X	X	
**DE**				X	
**GA**		X	X	X	X
**IA**		X	X	X	X
**IN**	X		X		
**KS**	X	X	X	X	X
**KY**	X	X	X	X	X
**LA**	X	X	X	X	X
**MD**				X	X
**MO**					X
**MS**		X	X	X	X
**NC**	X	X	X	X	X
**NE**		X	X		
**NH**	X	X	X	X	X
**NV**		X	X	X	X
**OH**			X	X	
**OK**	X	X	X	X	X
**OR**		X	X	X	
**SC**	X	X	X	X	X
**SD**	X	X	X		
**TN**	X	X	X	X	X
**TX**	X	X	X	X	X
**UT**					X
**WA**	X	X	X	X	X
**WY**				X	X

© 2025 Kranz AM et al. *JAMA Netw Open*.

eTable 4. **Estimated coefficients from IV model**

	Days from diagnosis to surgery	ED visit within 7 days of surgery	Hospitalization within 30 days of surgery	Medicaid Payment[a]
**Estimated coefficients (SE)**				
1[ASC]	-0.168* (0.029)	-0.223* (0.028)	-0.846* (0.170)	-0.269* (0.092)

Notes:
a. This sample is limited to 122,832 surgeries paid fee-for-service.
* p<0.01.
Abbreviations: ED, emergency department. ASC, ambulatory surgery center. SE, standard error. CI, confidence interval.

© 2025 Kranz AM et al. *JAMA Netw Open*.

eTable 5. Sensitivity test results: Instrumental Variable estimates of effects of receipt of care in an ASC on time to care, adverse outcomes, and payments for pediatric dental surgeries paid by Medicaid

	Days from diagnosis to surgery	ED visit within 7 days of surgery	Hospitalization within 30 days of surgery	Medicaid Payment[a]
**A. 2016-2020 Balance Panel (11 states)**				
**Estimated coefficients**				
1[ASC]	-0.199*	-0.162*	-0.780*	-0.328*
SE	(0.0206)	(0.0327)	(0.158)	(0.103)
**Margins**				
dy/dx	-10.61	-0.0027	-0.0022	-$750
SE	1.47	0.0006	0.0006	$263
P>|z|	P<0.001	P<0.001	P<0.001	0.004
**B. 2017-2020 Balance Panel (15 states)**				
**Estimated coefficients**				
1[ASC]	-0.175*	-0.179*	-0.938*	-0.326*
SE	(0.0364)	(0.0315)	(0.211)	(0.0930)
**Margins**				
dy/dx	-9.27	-0.0030	-0.0032	-$743
SE	2.22	0.0005	0.0012	$249
P>|z|	P<0.001	P<0.001	0.008	0.003

Notes:
a.This sample is limited to 122,832 surgeries paid fee-for-service.
* p<0.01.
Abbreviations: ED, emergency department. ASC, ambulatory surgery center. SE, standard error.

© 2025 Kranz AM et al. *JAMA Netw Open*.
