# Supplementary material for: Medicaid Payments and Outcomes for Pediatric Dental Surgical Procedures by Site of Care
Source: JAMA Netw Open. 2025 Oct 10;8(10):e2537081. doi: 10.1001/jamanetworkopen.2025.37081 (PMC12514629; doi:10.1001/jamanetworkopen.2025.37081)
Supplement: Supplement 2. — Data Sharing Statement [file jamanetwopen-e2537081-s002.pdf]

Data Sharing Statement

Kranz. Medicaid Payments and Outcomes for Pediatric Dental Surgical Procedures by Site of Care. *JAMA Netw Open*. Published October 10, 2025.
doi:10.1001/jamanetworkopen.2025.37081

Data
Data available: No

Additional Information
Explanation for why data not available: We cannot share these data due to restrictions present in our data use agreement (DUA). These data can be obtained for research purposes through an application process with the Research Data Assistance Center: https://resdac.org.
